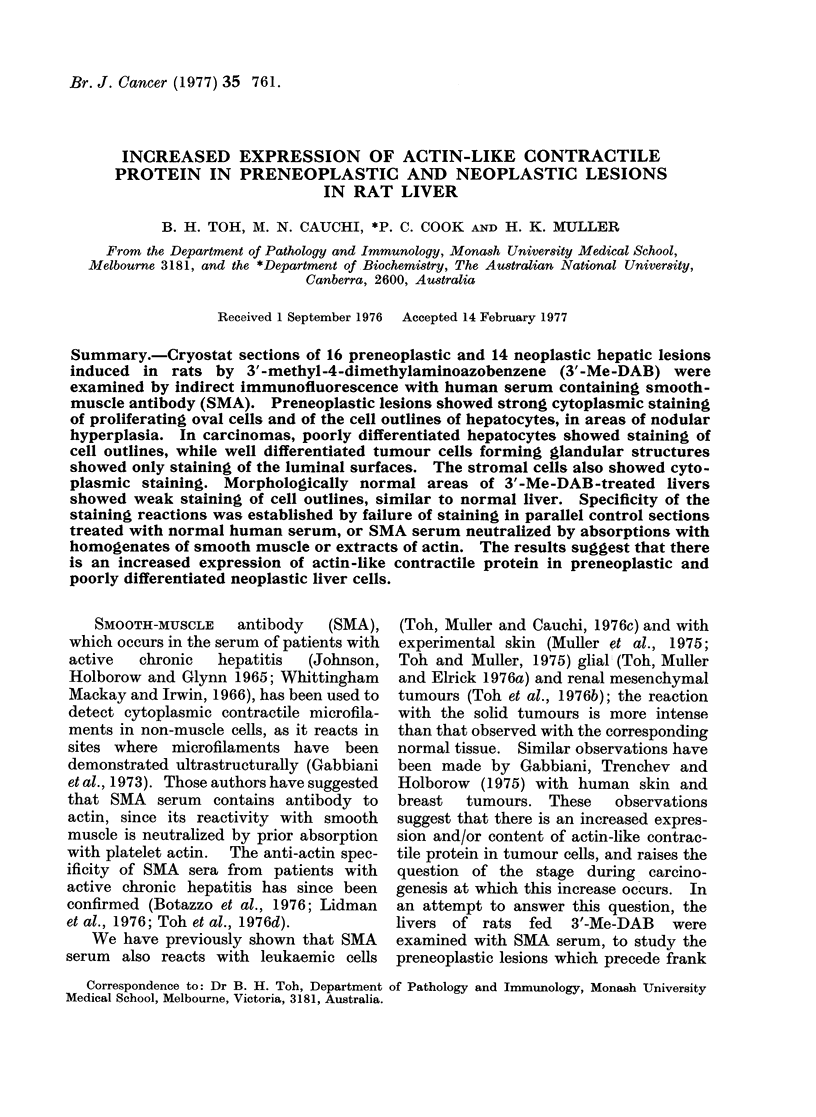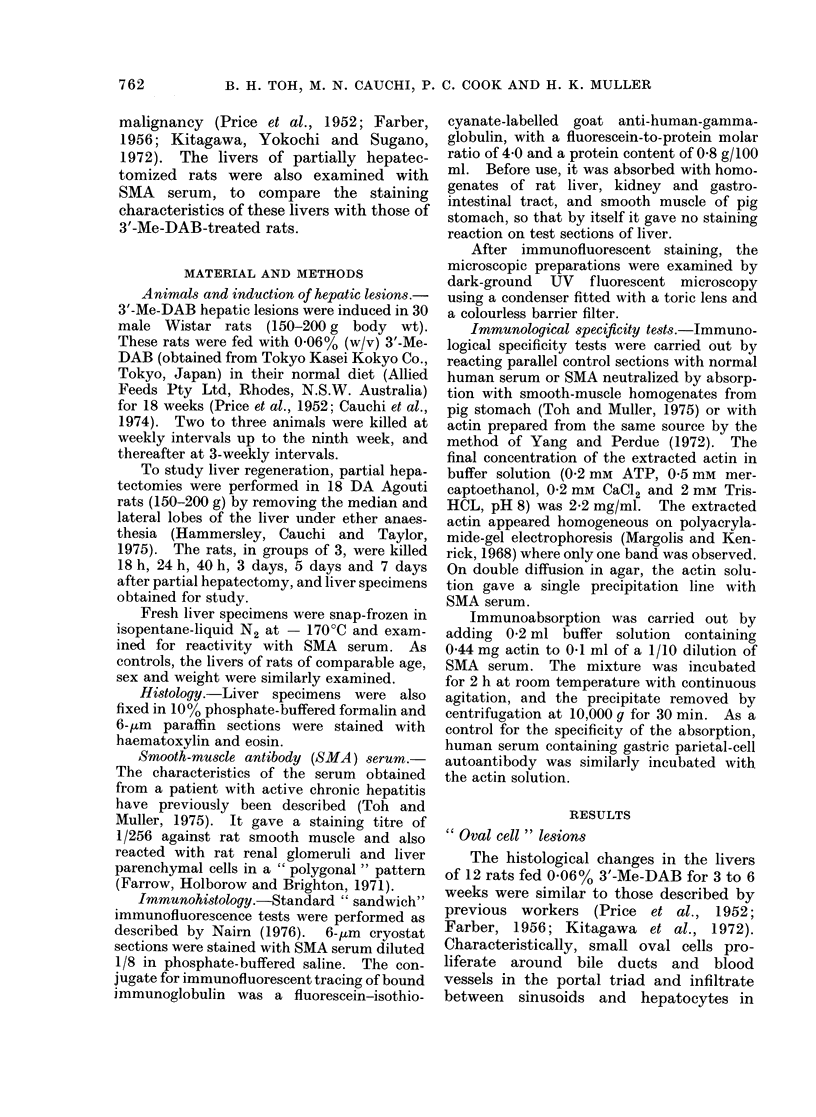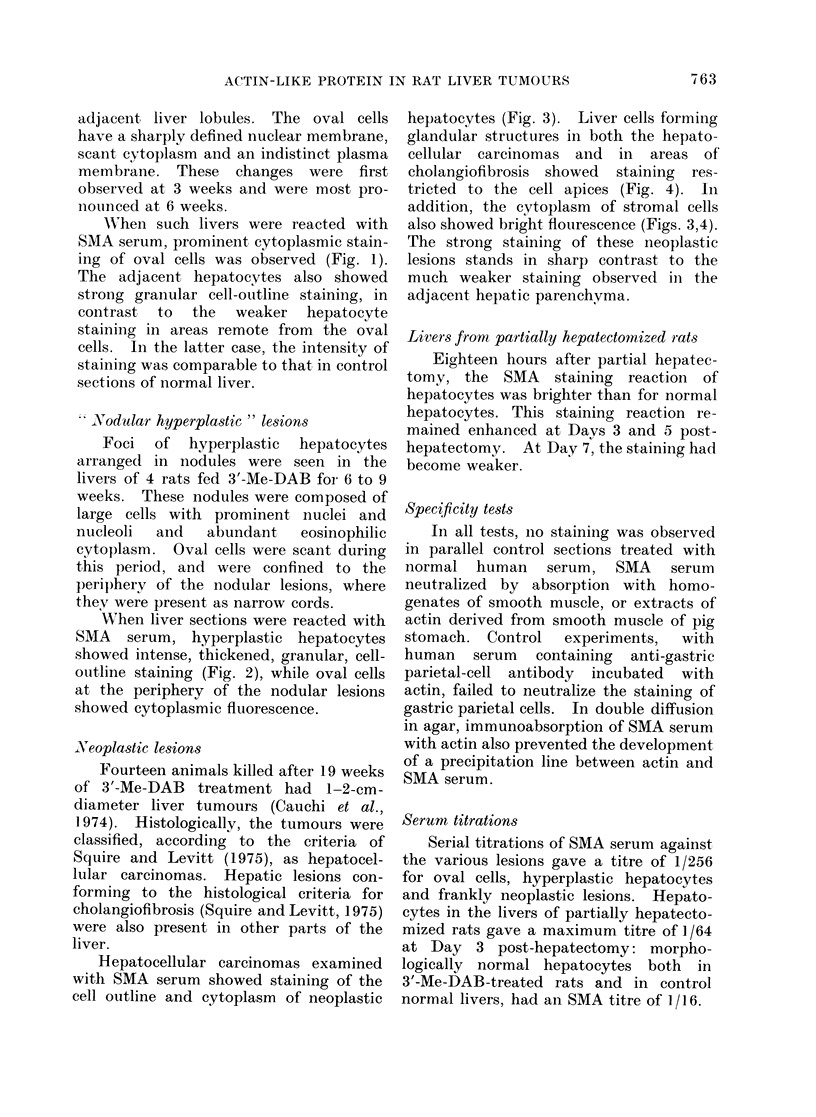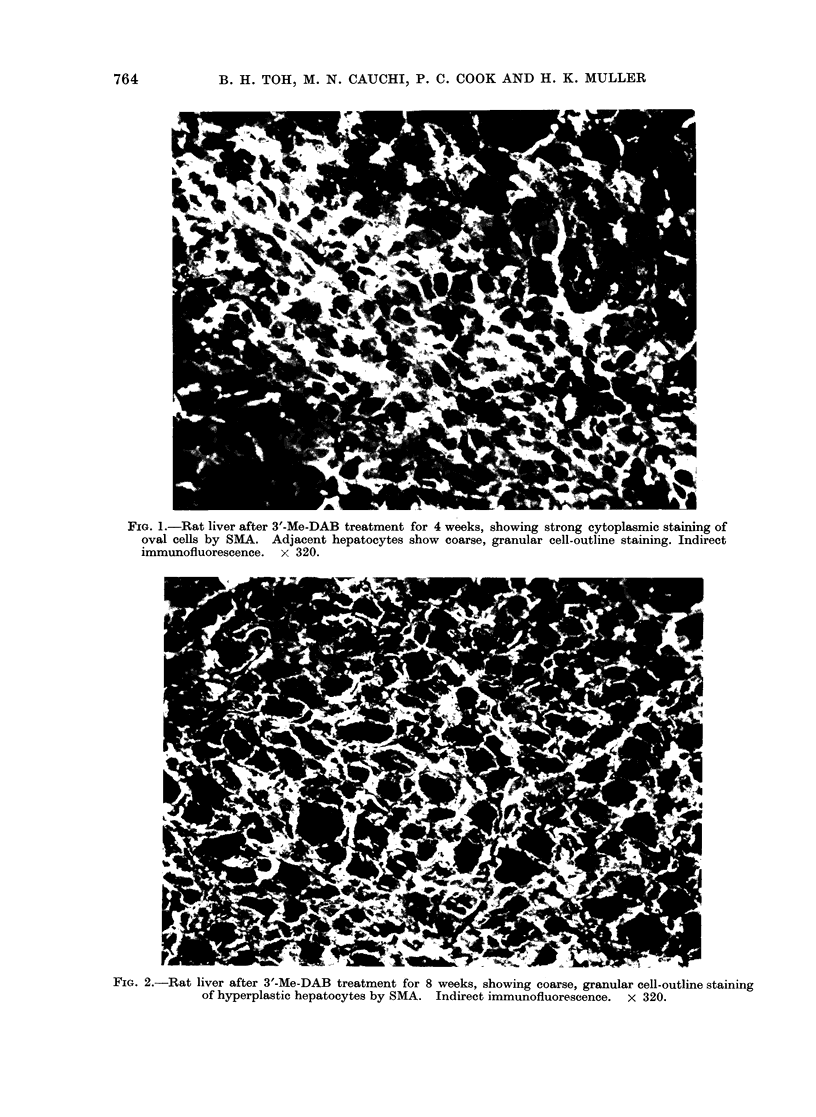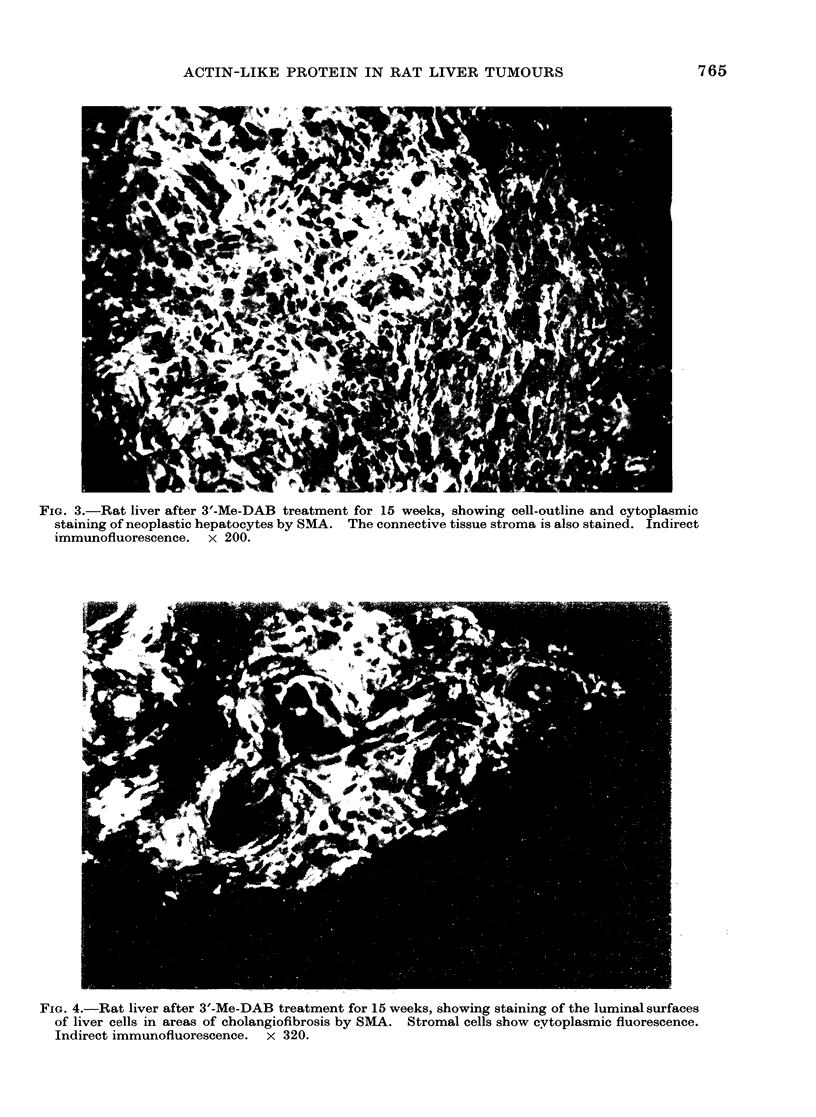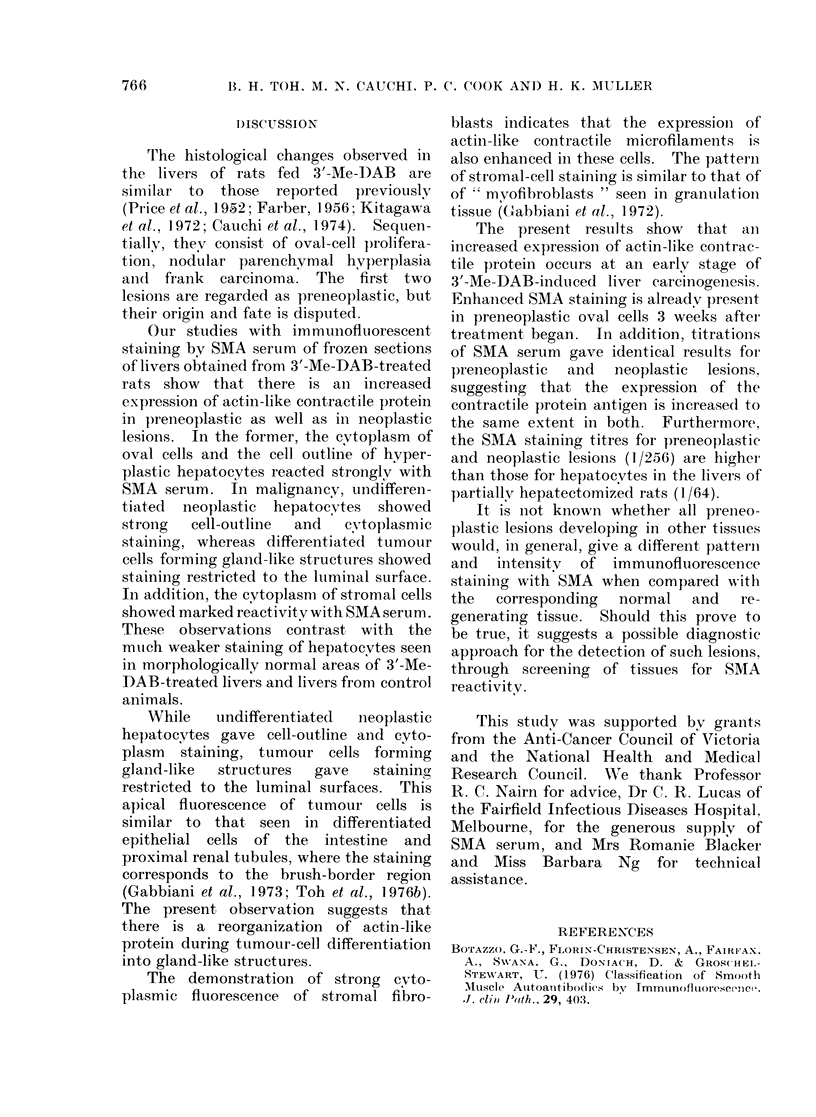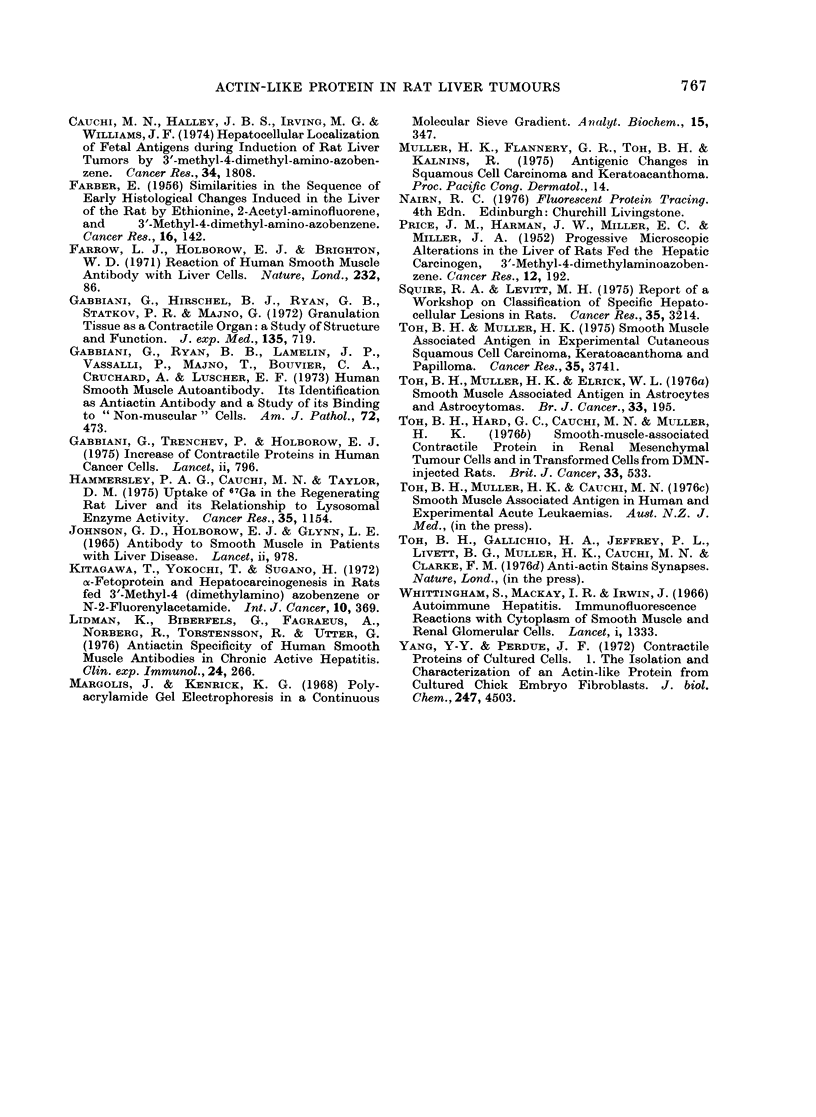# Increased expression of actin-like contractile protein in preneoplastic and neoplastic lesions in rat liver.

**DOI:** 10.1038/bjc.1977.117

**Published:** 1977-06

**Authors:** B. H. Toh, M. N. Cauchi, P. C. Cook, H. K. Muller

## Abstract

**Images:**


*Br. J. Cancer* (1977) **35** 761.

### INCREASED EXPRESSION OF ACTIN-LIKE CONTRACTILE PROTEIN IN PRENEOPLASTIC AND NEOPLASTIC LESIONS IN RAT LIVER

B. H. TOH, M. N. CAUCHI, *P. C. COOK AND H. K. MULLER

*From the Department of Pathology and Immunology, Monash University Medical School, Melbourne* 3181, *and the *Department of Biochemistry, The Australian National University, Canberra,* 2600, *Australia*

Received 1 September 1976 Accepted 14 February 1977

**Summary.—Cryostat sections of 16 preneoplastic and 14 neoplastic hepatic lesions induced in rats by 3′-methyl-4-dimethylaminoazobenzene (3′-Me-DAB) were examined by indirect immunofluorescence with human serum containing smooth-muscle antibody (SMA). Preneoplastic lesions showed strong cytoplasmic staining of proliferating oval cells and of the cell outlines of hepatocytes, in areas of nodular hyperplasia. In carcinomas, poorly differentiated hepatocytes showed staining of cell outlines, while well differentiated tumour cells forming glandular structures showed only staining of the luminal surfaces. The stromal cells also showed cytoplasmic staining. Morphologically normal areas of 3′-Me-DAB-treated livers showed weak staining of cell outlines, similar to normal liver. Specificity of the staining reactions was established by failure of staining in parallel control sections treated with normal human serum, or SMA serum neutralized by absorptions with homogenates of smooth muscle or extracts of actin. The results suggest that there is an increased expression of actin-like contractile protein in preneoplastic and poorly differentiated neoplastic liver cells.**

SMOOTH-MUSCLE antibody (SMA), which occurs in the serum of patients with active chronic hepatitis (Johnson, Holborow and Glynn 1965; Whittingham Mackay and Irwin, 1966), has been used to detect cytoplasmic contractile microfilaments in non-muscle cells, as it reacts in sites where microfilaments have been demonstrated ultrastructurally (Gabbiani *et al.*, 1973). Those authors have suggested that SMA serum contains antibody to actin, since its reactivity with smooth muscle is neutralized by prior absorption with platelet actin. The anti-actin specificity of SMA sera from patients with active chronic hepatitis has since been confirmed (Botazzo *et al.*, 1976; Lidman *et al.*, 1976; Toh *et al.*, 1976*d*).

We have previously shown that SMA serum also reacts with leukaemic cells (Toh, Muller and Cauchi, 1976*c*) and with experimental skin (Muller *et al.*, 1975; Toh and Muller, 1975) glial (Toh, Muller and Elrick 1976*a*) and renal mesenchymal tumours (Toh *et al.*, 1976*b*); the reaction with the solid tumours is more intense than that observed with the corresponding normal tissue. Similar observations have been made by Gabbiani, Trenchev and Holborow (1975) with human skin and breast tumours. These observations suggest that there is an increased expression and/or content of actin-like contractile protein in tumour cells, and raises the question of the stage during carcinogenesis at which this increase occurs. In an attempt to answer this question, the livers of rats fed 3′-Me-DAB were examined with SMA serum, to study the preneoplastic lesions which precede frank

Correspondence to: Dr B. H. Toh, Department of Pathology and Immunology, Monash University Medical School, Melbourne, Victoria, 3181, Australia.

malignancy (Price *et al.*, 1952; Farber, 1956; Kitagawa, Yokochi and Sugano, 1972). The livers of partially hepatectomized rats were also examined with SMA serum, to compare the staining characteristics of these livers with those of 3'-Me-DAB-treated rats.

### MATERIAL AND METHODS

*Animals and induction of hepatic lesions.*—3'-Me-DAB hepatic lesions were induced in 30 male Wistar rats (150–200 g body wt). These rats were fed with 0·06% (w/v) 3'-Me-DAB (obtained from Tokyo Kasei Kokyo Co., Tokyo, Japan) in their normal diet (Allied Feeds Pty Ltd, Rhodes, N.S.W. Australia) for 18 weeks (Price *et al.*, 1952; Cauchi *et al.*, 1974). Two to three animals were killed at weekly intervals up to the ninth week, and thereafter at 3-weekly intervals.

To study liver regeneration, partial hepatectomies were performed in 18 DA Agouti rats (150–200 g) by removing the median and lateral lobes of the liver under ether anaesthesia (Hammersley, Cauchi and Taylor, 1975). The rats, in groups of 3, were killed 18 h, 24 h, 40 h, 3 days, 5 days and 7 days after partial hepatectomy, and liver specimens obtained for study.

Fresh liver specimens were snap-frozen in isopentane-liquid $N_2$ at $-170°C$ and examined for reactivity with SMA serum. As controls, the livers of rats of comparable age, sex and weight were similarly examined.

*Histology.*—Liver specimens were also fixed in 10% phosphate-buffered formalin and 6-$\mu$m paraffin sections were stained with haematoxylin and eosin.

*Smooth-muscle antibody (SMA) serum.*—The characteristics of the serum obtained from a patient with active chronic hepatitis have previously been described (Toh and Muller, 1975). It gave a staining titre of 1/256 against rat smooth muscle and also reacted with rat renal glomeruli and liver parenchymal cells in a " polygonal " pattern (Farrow, Holborow and Brighton, 1971).

*Immunohistology.*—Standard " sandwich" immunofluorescence tests were performed as described by Nairn (1976). 6-$\mu$m cryostat sections were stained with SMA serum diluted 1/8 in phosphate-buffered saline. The conjugate for immunofluorescent tracing of bound immunoglobulin was a fluorescein–isothiocyanate-labelled goat anti-human-gamma-globulin, with a fluorescein-to-protein molar ratio of 4·0 and a protein content of 0·8 g/100 ml. Before use, it was absorbed with homogenates of rat liver, kidney and gastrointestinal tract, and smooth muscle of pig stomach, so that by itself it gave no staining reaction on test sections of liver.

After immunofluorescent staining, the microscopic preparations were examined by dark-ground UV fluorescent microscopy using a condenser fitted with a toric lens and a colourless barrier filter.

*Immunological specificity tests.*—Immunological specificity tests were carried out by reacting parallel control sections with normal human serum or SMA neutralized by absorption with smooth-muscle homogenates from pig stomach (Toh and Muller, 1975) or with actin prepared from the same source by the method of Yang and Perdue (1972). The final concentration of the extracted actin in buffer solution (0·2 mM ATP, 0·5 mM mercaptoethanol, 0·2 mM $CaCl_2$ and 2 mM Tris-HCL, pH 8) was 2·2 mg/ml. The extracted actin appeared homogeneous on polyacrylamide-gel electrophoresis (Margolis and Kenrick, 1968) where only one band was observed. On double diffusion in agar, the actin solution gave a single precipitation line with SMA serum.

Immunoabsorption was carried out by adding 0·2 ml buffer solution containing 0·44 mg actin to 0·1 ml of a 1/10 dilution of SMA serum. The mixture was incubated for 2 h at room temperature with continuous agitation, and the precipitate removed by centrifugation at 10,000 *g* for 30 min. As a control for the specificity of the absorption, human serum containing gastric parietal-cell autoantibody was similarly incubated with the actin solution.

### RESULTS

### " *Oval cell* " *lesions*

The histological changes in the livers of 12 rats fed 0·06% 3'-Me-DAB for 3 to 6 weeks were similar to those described by previous workers (Price *et al.*, 1952; Farber, 1956; Kitagawa *et al.*, 1972). Characteristically, small oval cells proliferate around bile ducts and blood vessels in the portal triad and infiltrate between sinusoids and hepatocytes in adjacent liver lobules. The oval cells have a sharply defined nuclear membrane, scant cytoplasm and an indistinct plasma membrane. These changes were first observed at 3 weeks and were most pronounced at 6 weeks.

When such livers were reacted with SMA serum, prominent cytoplasmic staining of oval cells was observed (Fig. 1). The adjacent hepatocytes also showed strong granular cell-outline staining, in contrast to the weaker hepatocyte staining in areas remote from the oval cells. In the latter case, the intensity of staining was comparable to that in control sections of normal liver.

*" Nodular hyperplastic " lesions*

Foci of hyperplastic hepatocytes arranged in nodules were seen in the livers of 4 rats fed 3′-Me-DAB for 6 to 9 weeks. These nodules were composed of large cells with prominent nuclei and nucleoli and abundant eosinophilic cytoplasm. Oval cells were scant during this period, and were confined to the periphery of the nodular lesions, where they were present as narrow cords.

When liver sections were reacted with SMA serum, hyperplastic hepatocytes showed intense, thickened, granular, cell-outline staining (Fig. 2), while oval cells at the periphery of the nodular lesions showed cytoplasmic fluorescence.

*Neoplastic lesions*

Fourteen animals killed after 19 weeks of 3′-Me-DAB treatment had 1–2-cm-diameter liver tumours (Cauchi *et al.*, 1974). Histologically, the tumours were classified, according to the criteria of Squire and Levitt (1975), as hepatocellular carcinomas. Hepatic lesions conforming to the histological criteria for cholangiofibrosis (Squire and Levitt, 1975) were also present in other parts of the liver.

Hepatocellular carcinomas examined with SMA serum showed staining of the cell outline and cytoplasm of neoplastic hepatocytes (Fig. 3). Liver cells forming glandular structures in both the hepatocellular carcinomas and in areas of cholangiofibrosis showed staining restricted to the cell apices (Fig. 4). In addition, the cytoplasm of stromal cells also showed bright flourescence (Figs. 3,4). The strong staining of these neoplastic lesions stands in sharp contrast to the much weaker staining observed in the adjacent hepatic parenchyma.

*Livers from partially hepatectomized rats*

Eighteen hours after partial hepatectomy, the SMA staining reaction of hepatocytes was brighter than for normal hepatocytes. This staining reaction remained enhanced at Days 3 and 5 post-hepatectomy. At Day 7, the staining had become weaker.

*Specificity tests*

In all tests, no staining was observed in parallel control sections treated with normal human serum, SMA serum neutralized by absorption with homogenates of smooth muscle, or extracts of actin derived from smooth muscle of pig stomach. Control experiments, with human serum containing anti-gastric parietal-cell antibody incubated with actin, failed to neutralize the staining of gastric parietal cells. In double diffusion in agar, immunoabsorption of SMA serum with actin also prevented the development of a precipitation line between actin and SMA serum.

*Serum titrations*

Serial titrations of SMA serum against the various lesions gave a titre of 1/256 for oval cells, hyperplastic hepatocytes and frankly neoplastic lesions. Hepatocytes in the livers of partially hepatectomized rats gave a maximum titre of 1/64 at Day 3 post-hepatectomy: morphologically normal hepatocytes both in 3′-Me-DAB-treated rats and in control normal livers, had an SMA titre of 1/16.

FIG. 1.—Rat liver after 3′-Me-DAB treatment for 4 weeks, showing strong cytoplasmic staining of oval cells by SMA. Adjacent hepatocytes show coarse, granular cell-outline staining. Indirect immunofluorescence. × 320.

FIG. 2.—Rat liver after 3′-Me-DAB treatment for 8 weeks, showing coarse, granular cell-outline staining of hyperplastic hepatocytes by SMA. Indirect immunofluorescence. × 320.

FIG. 3.—Rat liver after 3′-Me-DAB treatment for 15 weeks, showing cell-outline and cytoplasmic staining of neoplastic hepatocytes by SMA. The connective tissue stroma is also stained. Indirect immunofluorescence. × 200.

FIG. 4.—Rat liver after 3′-Me-DAB treatment for 15 weeks, showing staining of the luminal surfaces of liver cells in areas of cholangiofibrosis by SMA. Stromal cells show cytoplasmic fluorescence. Indirect immunofluorescence. × 320.

### DISCUSSION

The histological changes observed in the livers of rats fed 3′-Me-DAB are similar to those reported previously (Price *et al.*, 1952; Farber, 1956; Kitagawa *et al.*, 1972; Cauchi *et al.*, 1974). Sequentially, they consist of oval-cell proliferation, nodular parenchymal hyperplasia and frank carcinoma. The first two lesions are regarded as preneoplastic, but their origin and fate is disputed.

Our studies with immunofluorescent staining by SMA serum of frozen sections of livers obtained from 3′-Me-DAB-treated rats show that there is an increased expression of actin-like contractile protein in preneoplastic as well as in neoplastic lesions. In the former, the cytoplasm of oval cells and the cell outline of hyperplastic hepatocytes reacted strongly with SMA serum. In malignancy, undifferentiated neoplastic hepatocytes showed strong cell-outline and cytoplasmic staining, whereas differentiated tumour cells forming gland-like structures showed staining restricted to the luminal surface. In addition, the cytoplasm of stromal cells showed marked reactivity with SMA serum. These observations contrast with the much weaker staining of hepatocytes seen in morphologically normal areas of 3′-Me-DAB-treated livers and livers from control animals.

While undifferentiated neoplastic hepatocytes gave cell-outline and cytoplasm staining, tumour cells forming gland-like structures gave staining restricted to the luminal surfaces. This apical fluorescence of tumour cells is similar to that seen in differentiated epithelial cells of the intestine and proximal renal tubules, where the staining corresponds to the brush-border region (Gabbiani *et al.*, 1973; Toh *et al.*, 1976*b*). The present observation suggests that there is a reorganization of actin-like protein during tumour-cell differentiation into gland-like structures.

The demonstration of strong cytoplasmic fluorescence of stromal fibroblasts indicates that the expression of actin-like contractile microfilaments is also enhanced in these cells. The pattern of stromal-cell staining is similar to that of of " myofibroblasts " seen in granulation tissue (Gabbiani *et al.*, 1972).

The present results show that an increased expression of actin-like contractile protein occurs at an early stage of 3′-Me-DAB-induced liver carcinogenesis. Enhanced SMA staining is already present in preneoplastic oval cells 3 weeks after treatment began. In addition, titrations of SMA serum gave identical results for preneoplastic and neoplastic lesions, suggesting that the expression of the contractile protein antigen is increased to the same extent in both. Furthermore, the SMA staining titres for preneoplastic and neoplastic lesions (1/256) are higher than those for hepatocytes in the livers of partially hepatectomized rats (1/64).

It is not known whether all preneoplastic lesions developing in other tissues would, in general, give a different pattern and intensity of immunofluorescence staining with SMA when compared with the corresponding normal and regenerating tissue. Should this prove to be true, it suggests a possible diagnostic approach for the detection of such lesions, through screening of tissues for SMA reactivity.

This study was supported by grants from the Anti-Cancer Council of Victoria and the National Health and Medical Research Council. We thank Professor R. C. Nairn for advice, Dr C. R. Lucas of the Fairfield Infectious Diseases Hospital, Melbourne, for the generous supply of SMA serum, and Mrs Romanie Blacker and Miss Barbara Ng for technical assistance.

### REFERENCES

BOTAZZO, G.-F., FLORIN-CHRISTENSEN, A., FAIRFAX, A., SWANA, G., DONIACH, D. & GROSCHEL-STEWART, U. (1976) Classification of Smooth Muscle Autoantibodies by Immunofluorescence. *J. clin Path.*, **29**, 403.

CAUCHI, M. N., HALLEY, J. B. S., IRVING, M. G. & WILLIAMS, J. F. (1974) Hepatocellular Localization of Fetal Antigens during Induction of Rat Liver Tumors by 3′-methyl-4-dimethyl-amino-azobenzene. *Cancer Res.*, **34,** 1808.

FARBER, E. (1956) Similarities in the Sequence of Early Histological Changes Induced in the Liver of the Rat by Ethionine, 2-Acetyl-aminofluorene, and 3′-Methyl-4-dimethyl-amino-azobenzene. *Cancer Res.*, **16,** 142.

FARROW, L. J., HOLBOROW, E. J. & BRIGHTON, W. D. (1971) Reaction of Human Smooth Muscle Antibody with Liver Cells. *Nature, Lond.*, **232,** 86.

GABBIANI, G., HIRSCHEL, B. J., RYAN, G. B., STATKOV, P. R. & MAJNO, G. (1972) Granulation Tissue as a Contractile Organ: a Study of Structure and Function. *J. exp. Med.*, **135,** 719.

GABBIANI, G., RYAN, B. B., LAMELIN, J. P., VASSALLI, P., MAJNO, T., BOUVIER, C. A., CRUCHARD, A. & LUSCHER, E. F. (1973) Human Smooth Muscle Autoantibody. Its Identification as Antiactin Antibody and a Study of its Binding to " Non-muscular " Cells. *Am. J. Pathol.*, **72,** 473.

GABBIANI, G., TRENCHEV, P. & HOLBOROW, E. J. (1975) Increase of Contractile Proteins in Human Cancer Cells. *Lancet*, ii, 796.

HAMMERSLEY, P. A. G., CAUCHI, M. N. & TAYLOR, D. M. (1975) Uptake of $^{67}$Ga in the Regenerating Rat Liver and its Relationship to Lysosomal Enzyme Activity. *Cancer Res.*, **35,** 1154.

JOHNSON, G. D., HOLBOROW, E. J. & GLYNN, L. E. (1965) Antibody to Smooth Muscle in Patients with Liver Disease. *Lancet*, ii, 978.

KITAGAWA, T., YOKOCHI, T. & SUGANO, H. (1972) α-Fetoprotein and Hepatocarcinogenesis in Rats fed 3′-Methyl-4 (dimethylamino) azobenzene or N-2-Fluorenylacetamide. *Int. J. Cancer*, **10,** 369.

LIDMAN, K., BIBERFELS, G., FAGRAEUS, A., NORBERG, R., TORSTENSSON, R. & UTTER, G. (1976) Antiactin Specificity of Human Smooth Muscle Antibodies in Chronic Active Hepatitis. *Clin. exp. Immunol.*, **24,** 266.

MARGOLIS, J. & KENRICK, K. G. (1968) Polyacrylamide Gel Electrophoresis in a Continuous Molecular Sieve Gradient. *Analyt. Biochem.*, **15,** 347.

MULLER, H. K., FLANNERY, G. R., TOH, B. H. & KALNINS, R. (1975) Antigenic Changes in Squamous Cell Carcinoma and Keratoacanthoma. *Proc. Pacific Cong. Dermatol.*, 14.

NAIRN, R. C. (1976) *Fluorescent Protein Tracing.* 4th Edn. Edinburgh: Churchill Livingstone.

PRICE, J. M., HARMAN, J. W., MILLER, E. C. & MILLER, J. A. (1952) Progessive Microscopic Alterations in the Liver of Rats Fed the Hepatic Carcinogen, 3′-Methyl-4-dimethylaminoazobenzene. *Cancer Res.*, **12,** 192.

SQUIRE, R. A. & LEVITT, M. H. (1975) Report of a Workshop on Classification of Specific Hepatocellular Lesions in Rats. *Cancer Res.*, **35,** 3214.

TOH, B. H. & MULLER, H. K. (1975) Smooth Muscle Associated Antigen in Experimental Cutaneous Squamous Cell Carcinoma, Keratoacanthoma and Papilloma. *Cancer Res.*, **35,** 3741.

TOH, B. H., MULLER, H. K. & ELRICK, W. L. (1976*a*) Smooth Muscle Associated Antigen in Astrocytes and Astrocytomas. *Br. J. Cancer.*, **33,** 195.

TOH, B. H., HARD, G. C., CAUCHI, M. N. & MULLER, H. K. (1976*b*) Smooth-muscle-associated Contractile Protein in Renal Mesenchymal Tumour Cells and in Transformed Cells from DMN-injected Rats. *Brit. J. Cancer*, **33,** 533.

TOH, B. H., MULLER, H. K. & CAUCHI, M. N. (1976*c*) Smooth Muscle Associated Antigen in Human and Experimental Acute Leukaemias. *Aust. N.Z. J. Med.*, (in the press).

TOH, B. H., GALLICHIO, H. A., JEFFREY, P. L., LIVETT, B. G., MULLER, H. K., CAUCHI, M. N. & CLARKE, F. M. (1976*d*) Anti-actin Stains Synapses. *Nature, Lond.*, (in the press).

WHITTINGHAM, S., MACKAY, I. R. & IRWIN, J. (1966) Autoimmune Hepatitis. Immunofluorescence Reactions with Cytoplasm of Smooth Muscle and Renal Glomerular Cells. *Lancet*, i, 1333.

YANG, Y-Y. & PERDUE, J. F. (1972) Contractile Proteins of Cultured Cells. 1. The Isolation and Characterization of an Actin-like Protein from Cultured Chick Embryo Fibroblasts. *J. biol. Chem.*, **247,** 4503.