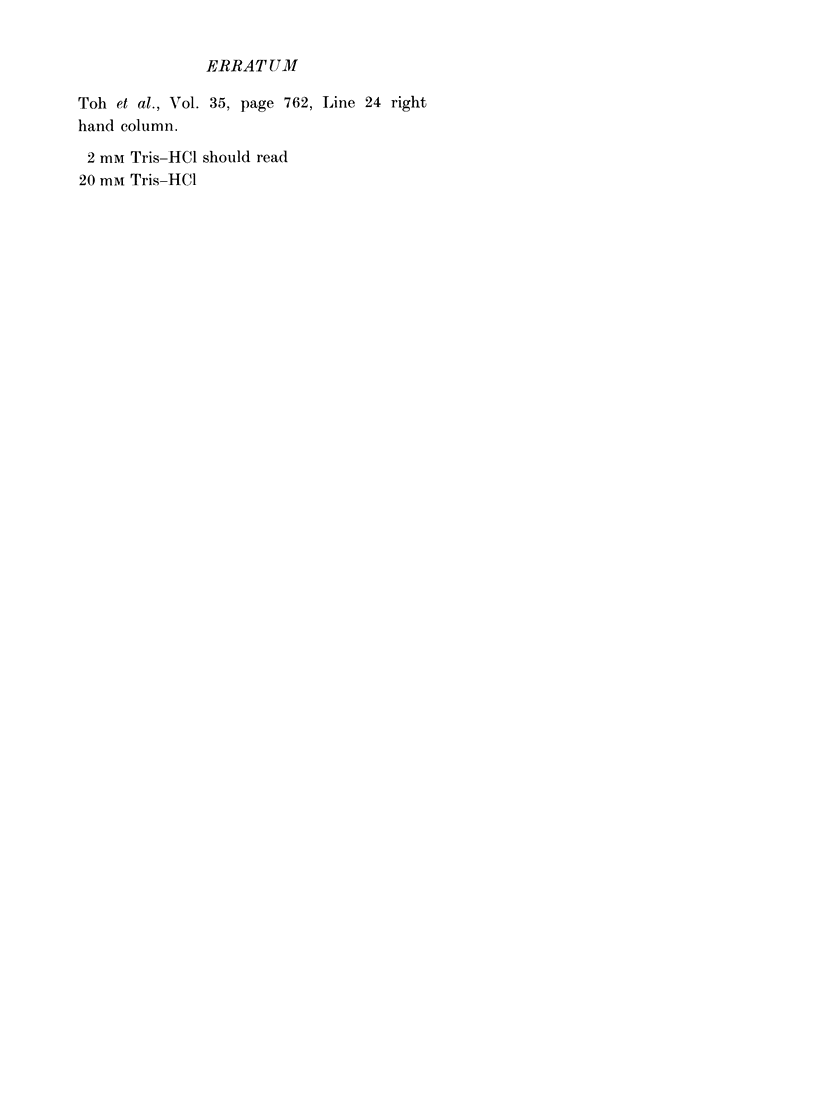# Erratum

**Published:** 1977-06

**Authors:** 


### *ERRATUM*

Toh *et al.*, Vol. 35, page 762, Line 24 right hand column.

2 mM Tris–HCl should read
20 mM Tris–HCl